# Measurement and identification of relative poverty level of pastoral areas: an analysis based on spatial layout

**DOI:** 10.1007/s11356-022-21717-6

**Published:** 2022-07-08

**Authors:** Haiying Lin, Youhan Gao, Tianqi Zhu, Huayuan Wu, Pengshen Hou, Wenlong Li, Shuxia Hou, Muhammad Umer Arshad

**Affiliations:** 1 Business School, Inner Mongolia University of Finance and Economics, Hohhot, 010070 China; 2 Graduate School Inner, Mongolia University of Finance and Economics, Hohhot, 010070 China; 3 Inner Mongolia Agricultural University, Vocational and Technical College, Baotou, 014109 China; 4 Resources, Environment and Economics School, Inner Mongolia University of Finance and Economics, Hohhot, 010070 China; 5 Inner Mongolia Hondar College of Arts and Science, Hohhot, Inner Mongolia China

**Keywords:** Quality of rural life (QRL), Relative poverty, Pastoral areas, Spatial layout, China

## Abstract

Pastoral areas are the key difficulty in China’s pursuit of common prosperity and a key region for China to build the northern ecological safety barrier and to realize the Two Centenary Goals. It is of great significance to scientifically evaluate the quality of rural life (QRL), measure the relative poverty level (RPL), and identify the relatively poor areas, making it possible to dock poverty elimination with rural revitalization. Based on the socio-economic data of 18 pastoral areas in Inner Mongolia, this paper draws on spatial layout theory to evaluate QRL and measures RPL by the natural breakpoint method and then identifies the relatively poor areas in Inner Mongolia. The results show that (1) the QRLs of pastoral areas in Inner Mongolia were unbalanced and highly polarized. The mean score of QRLs was 0.2598. Eleven (61.11%) of the counties/banners had a QRL smaller than the mean score. On the spatial layout of QRLs, the western areas were stronger than the central areas. High QRL counties/banners are mainly concentrated in the western region. In the central region, the QRLs were very fragmented, falling onto all five levels. (2) The pastoral areas in Inner Mongolia differed significantly in RPL. The mean score of RPL stood at 0.3788. Nine counties/banners (50%) had an RPL greater than the mean. Contrary to the spatial layout features of QRLs, the central pastoral areas in Inner Mongolia had stronger RPLs than the eastern ones. High RPL counties/banners are mostly clustered in the central region. The spatial layout of RPLs is relatively reasonable in the central region: the RPLs decreased gradually from Dorbod Banner. (3) Nearly 45% of the pastoral areas in central and western Inner Mongolia face serious relative poverty and a high risk of returning to poverty. Eight counties/banners (45%) were identified as high composite relative poverty areas. From spatial layout, the composite relatively poor counties/banners clustered clearly, mainly in the western region. Finally, this paper establishes a warning mechanism against large-scale returning to poverty, aiming to lower composite RPL. The research results provide empirical reference and implementation path for consolidating the results of poverty eradication and facilitating rural revitalization.

## Introduction

As of 2020, China had lifted all its 98.99 million absolute-poverty-strike rural residents out of poverty. The eradication of absolute poverty and region-wide poverty marks the beginning of the post-poverty reduction era. But China still faces prominent problems like poverty-returning and relative poverty after the elimination of absolute poverty. According to the Chinese president, China needs to complete a historical transformation of its poverty alleviation work from focusing on the eradication of absolute poverty to solving relative poverty. The ruling party of China clearly stated that to consolidate the achievements of poverty elimination, an important task is to establish a long-acting mechanism that solves relative poverty. Unfortunately, the poverty-returning rate remains high in China, reaching 20% in ethnic minority areas (Aaberge and Brandolini 2015).

The ruling party also pledged to attach importance to relative poverty and its identification standards, realize effective governance of relative poverty, and seamlessly dock poverty elimination with rural revitalization. Upon entering the post-poverty reduction era, China, moving away from the survival-threatening absolute poverty, embarks on the journey towards the grand goal of common prosperity. To achieve common prosperity, it is a must to govern relative poverty. With the in-depth research on relative poverty, more and more attention has been paid to the measurement of the relative poverty level (RPL), identifying relatively poor areas, and preventing the marginal population from returning to poverty. The research in these aspects is of great significance to solving relative poverty and poverty-returning issues.

The existing studies on poverty concentrate on the measurement of multidimensional poverty. When it comes to the impactors, causes, and governance of relative poverty, there are insufficient research efforts to measure RPL from the angle of the quality of rural life (QRL) and to identify relatively poor areas comprehensively.

Relative poverty has been explored deeply since scholars realized that it challenges absolute poverty as the standard for identifying poverty-stricken populations (Ali 1998). Poverty eradication is the common vision and goal of the international community, including international organizations and all countries. Developing countries mainly strive to eradicate absolute poverty (Alkire and Foster 2011), while developed countries mostly attempt to eliminate relative poverty (Alkire et al. 2014; Alkire and Santos 2010, 2014). Relative poverty means the income is relatively lower than the national average household income (Sen 1983). Absolute poverty largely originates from the lack of material wealth, while relative poverty comes from the imbalance between development and distribution. In this sense, absolute poverty can be eliminated, but relative poverty can only be alleviated rather than eliminated. Whether in developed or developing countries, relative poverty will persist for a long time (Sen 1983). Poverty causes unbalanced development among regions and affects regional peace and stability. The outbreak of Covid-19 caused the loss of about 114 million jobs worldwide and threw around 120 million people into extreme poverty, posing a huge threat to the global poverty governance. Facing various challenges, eliminating absolute poverty, and reducing relative poverty have become an important issue for the development of human society (Antonella and Giovanni 2022).

Relative poverty means the average income is below the mean of national household income (Atkinson et al. 2002). Adam Smith was the first to study relative poverty in his book The Wealth of Nations. Friedman (1965) clearly defined relative poverty as the poverty exposed by the changing living standards. Amartya Sen (Atkinson et al. 2002; Banerjee and Duflo 2014) held that poverty is capability deprivation and inspired multidimensional poverty indices based on income poverty, unveiling the era of multidimensional poverty measurement (Banzhaf et al. 2014; Berihuete et al. 2018; Chen et al. 2018, 2021; Christiaensen and Todo 2014). Subsequently, income level was taken as the relative poverty standard, and relative poverty was measured by the expenditure method and the asset index method (Decancq and Lugo 2012).

Some scholars focused on the objects of relative poverty. In the identification of poor families, the United Nations Development Programme (UNDP) (Drewnowski and Specter 2004) treated per capita income, life expectancy, and literacy rate as effective indices of poor families (Duckett 1998; Fujii 2017; Gao and Zhai 2012; Gao et al. 2011; Gazeley and Verdon 2014; Ge et al. 2017). In the identification of poor areas, Alkire et al. (Gao and Zhai 2012) delegated the power of poverty identification to village, township, and county levels and designed local approaches that identify relatively poor people according to local conditions.

Relative poverty is dynamic rather than fixed. With the improvement of poverty standards, new poor households have emerged, and the rural population, who are no longer poor, are weakly vulnerable and easy to return to poverty, forming new cumulative poverty (Gustafsson and Sai 2020). Huang and Cox (Foster 1998; Silverman and Holtyn 2016) divided the causes of relative poverty into subjective factors, economic factors, institutional factors, and environmental factors and presented multiple countermeasures, such as improving and innovating the social assistance and security mechanism and training vulnerable farmers.

Banerjee and Duflo (Keswell and Carter 2014), winners of the Nobel Memorial Prize in Economic Sciences 2019, highlighted in their book *Poor Economics: A Radical Rethinking of the Way to Fight Global Poverty* that the essence of poverty is that the poor are unable to jump out of the poverty trap, and early prevention plus follow-up risk warning enables humans to rid poverty in the long term. Among the various international organizations, the World Bank emphasizes the standards for absolute poverty. By contrast, The Organisation for Economic Co-operation and Development (OECD) adopts a set of relative poverty standards but does not set specific goals and progress. The European Union became the only international organization to clarify the goals of mitigating relative poverty, as it published the policy document Europe 2020 in 2010. Yet this document is concerned with the poverty-returning risk.

The poverty-returning warning mechanism is crucial to the prevention of largescale returning to poverty. The relevant studies mainly tackle two aspects: poverty-returning causes and poverty-returning governance. The primary causes of poverty-returning are changes in the eco-environment, assets, diseases, and abilities (Kim 2016; Li et al. 2022). The existing governance measures for poverty-returning are mainly mitigation and prevention methods, relying on government and organizations, as well as poverty alleviation systems and policies (Li et al. 2022) (Liang and Hui 2016). The relevant scholars have examined the possible reasons for households to return to poverty, recognized poverty-returning as an objective fact, and found that ethnic minority residents more easily return to poverty (Aaberge and Brandolini 2015). Various causes have been identified for poverty-returning (Liu and Xu 2016): people may return to poverty due to institutions and policies (Liu and Li 2020), resources and environment (Luan and Liu 2021), disasters and risks (Shaukat Ali 1995), and abilities and habits (Ma et al. 2019).

The warning mechanism for poverty-returning is also a research hotspot. To curb the return to poverty, the relevant scholars have analyzed the poverty-returning warning mechanism from such dimensions as education and employment, medical security, eco-environment construction, and subject participation (Malise 2006; Martilla et al. 1997; Mondal and Shitan 2014; Nie et al. 2021; Nurkse 1966). The early prevention of poverty-returning is a key link to poverty governance. A perfect warning mechanism of poverty-returning risk, coupled with follow-up support to those being lifted out of poverty, can safeguard their sustainable livelihood and minimize the poverty-returning probability (Pokhriyal and Jacques 2017; Strier 2009). Therefore, it is important to build an all-dimensional warning mechanism for poverty-returning, after reasonably recognizing and measuring RPL. However, academia has not fully or systematically studied such a mechanism.

Despite exploring the governance of relative poverty, the existing studies have several gaps: (1) most of them only measure absolute poverty and multidimensional poverty and failed to measure the RPL in special relatively poor areas (e.g., the pastoral areas in Inner Mongolia). In particular, the RPL has not been measured from the perspective of the life quality in rural pastoral areas. (2) The previous literature mostly tackles development-oriented poverty alleviation and reduction, as well as the governance of those who have returned to poverty. The relatively poor areas and objects have not been identified comprehensively before poverty-returning. (3) Relative poverty governance has only been quantitatively analyzed in terms of time. No report measures the RPL of pastoral areas or identifies composite relatively poor areas from the perspective of spatial layout.

Inner Mongolia, a borderland in China, has many famous pastoral areas. The unique ethnicity, culture, and ecology make relative poverty a prominent issue in the pastoral areas of Inner Mongolia. Pastoral areas are the key difficulty in China’s pursuit of common prosperity and a key region for China to build the northern ecological safety barrier and realize the Two Centenary Goals. It is of great significance to scientifically evaluate QRL, measure the relative poverty level RPL, and identify the relatively poor areas, making it possible to dock poverty elimination with rural revitalization. In addition, this study can enrich the theory of poverty governance and improve the measurement method of the relative poverty level.

Based on the socio-economic data of 18 pastoral areas in Inner Mongolia, this paper draws on spatial layout theory to discuss several important issues by natural breakpoint method, using ArcGIS 10.8. (1) Are there significant differences between the pastoral areas in QRL? What are the features of the spatial layout of QRLs? (2) Are there significant differences between the pastoral areas in RPL? What are the features of the spatial layout of RPLs? (3) What is the way to identify composite relatively poor areas in the pastoral areas? Are there spatial differences between composite RPLs? If yes, what are the causes of these differences? What are the features of the spatial layout of composite RPLs?

The remainder of this paper is organized as follows: "Overview of Study Area" overviews the study area; "Research Design" reports the research design, including data sources and preprocessing selection of research methods, and selection of variables; "Results Analysis" analyzes the empirical results; "Discussion" presents a general discussion; "Conclusions and Suggestions" summarizes the conclusions and proposes several suggestions for policy-makers.

## Overview of study area

Dominated by a temperate continental monsoon climate, Inner Mongolia features limited and uneven rainfall, abundant sunshine, and dramatic changes between cold and hot seasons. Lying in the north of China, Inner Mongolia is bordered by Mongolia and Russia and administers 9 prefectures and 3 leagues. The total area of Inner Mongolia is 1.183 million km2, 12.3% of the total landmass of China. Grasslands (88 million hectares) take up 49.59% of the total area of Inner Mongolia, making the autonomous region one of the four largest farming and pastoral regions in China. In 2020, the gross domestic product (GDP) of Inner Mongolia was worth 1.735982 trillion yuan, about 1.7% of China’s total GDP. The per capita GDP stood at 72.062 yuan. Specifically, the primary industry in the region outputted a value of 202.512 billion yuan, accounting for 2.6% of the national primary industry output; the agricultural output of the region was 169.9 billion yuan, 2.4% of China’s total agricultural output. The above data were collected from the statistical bulletin released by the Government of Inner Mongolia in 2020 (https://www.nmg.gov.cn/).

Apart from the general feature of county-level administrative regions in China, the counties/banners in Inner Mongolia have a unique regional feature: the integration between farming and animal husbandry (Ravallion and S. 2011). Currently, Inner Mongolia administers 33 pastoral counties/banners (hereinafter referred to as pastoral areas). These pastoral areas occupy a landmass of 816,300 km2, accounting for 69.00% of the total area of Inner Mongolia. In 2020, the population of the pastoral areas reached 5.9047 million, about 21.20% of the total population of Inner Mongolia, and the grassland area amounted to 816.2002 million mu.

On August 27, 2021, the Office of the Central Rural Work Leading Group, and National Rural Revitalization Administration reassessed the 832 poor counties/banners in 22 provincial administrative regions and identified 160 counties/banners in 8 provincial administrative regions as the key support objects of national rural revitalization campaign. The 31 poor counties/banners in Inner Mongolia were reduced to 10 support counties/banners: Oroqen Autonomous Banner (Hulunbuir Prefecture); Horqin Right Middle Banner, Jalaid Banner, and Horqin Right Front Banner (Hinggan League); Bairin Left Banner (Chifeng Prefecture); Hure Banner (Tongliao Prefecture); Plain and Bordered White Banner (Xilin Gol League); Shangdu County, Huade County, and Dorbod Banner (Ulanqab Prefecture).

In the above 10 counties/banners, 186,800 people of 8.23 households have been lifted out of poverty, taking up 22.19% and 22% of the total number of people and households getting out of poverty in Inner Mongolia, respectively; 8,400 people of 4,100 households are prone to return to poverty, accounting for 27.23% and 26.9% of the total number of people and households on the edge of poverty in Inner Mongolia, respectively. Among them, Bairin Left Banner, Plain and Bordered White Banner, and Dorbod Banner belong to pastoral areas. The above data come from the Rural Revitalization Administration of Inner Mongolia (http://fpb.nmg.gov.cn).

Considering data availability and statistical limitation, this paper chooses to study the 18 pastoral areas in central and western Inner Mongolia. With a total area of 565,400km2 (69.26% of the total areas of all pastoral areas in Inner Mongolia), the studied pastoral areas are home to 1.6406 million people (27.78% of the total population in all pastoral areas in Inner Mongolia) and own a total of 513.9472 million mu (62.97% of the total grassland area in all pastoral areas in Inner Mongolia).

## Research design

### Data sources and preprocessing

The research data include socioeconomic data, natural basic data, and spatial data. The socioeconomic data and natural basic data were collected from the Inner Mongolia Statistical Yearbook 2021, the statistical bulletins of different prefectures/leagues, the statistical bulletins of different counties/banners, and the meteorological department. The spatial vector data were acquired from the National Platform for Common Geospatial Information Services (https://www.ngcc.cn/ngcc/). To ensure the continuity and scientificity of research data, the missing data were interpolated or linearly interpolated with the data from nearby stations.

The original data were normalized to eliminate the influence of numerical size and dimension on the results. The data preprocessing includes the following steps: (1) range normalization of the original data; (2) assigning the weight to each index using the coefficient of variation (COV), a popular tool for spatial difference analysis, aiming to reduce the influence of subjective factors on analysis results; (3) weight calculation. The specific formulas are as follows:

Range normalization of positive indices:

1 $${Y}_{\mathrm{i}}=\frac{{X}_{\mathrm{i}}-M\mathrm{in}\left({X}_{\mathrm{i}}\right)}{Max\left({X}_{\mathrm{i}}\right)-M\mathrm{in}\left({X}_{\mathrm{i}}\right)}$$

Range normalization of negative indices:

2 $${Y}_{\mathrm{i}}=\frac{M\mathrm{ax}\left({X}_{\mathrm{i}}\right)-{X}_{\mathrm{i}}}{M\mathrm{ax}\left({X}_{\mathrm{i}}\right)-M\mathrm{in}\left({X}_{\mathrm{i}}\right)}$$

COV:

3 $${\delta }_{\mathrm{i}}=\frac{{SD}_{\mathrm{i}}}{{\overline{Y} }_{\mathrm{i}}}$$

Index weight:

4 $${W}_{\mathrm{i}}=\frac{{\delta }_{\mathrm{i}}}{\sum\limits_{\mathrm{i}=1}^{\mathrm{n}}{\delta }_{\mathrm{i}}}$$

where ${X}_{\mathrm{i}}$ and ${Y}_{\mathrm{i}}$ are the original and normalized values of index *i* of a county/banner, respectively; $M\mathrm{ax}\left({X}_{\mathrm{i}}\right)$ and $M\mathrm{in}\left({X}_{\mathrm{i}}\right)$ are the maximum and minimum of index i, respectively; ${SD}_{\mathrm{i}}$ and ${\overline{Y} }_{\mathrm{i}}$ are the standard deviation and mean of index *i*, respectively.

### Selection of methods and variables

#### QRL calculation

QRL evaluation provides the basic data for subsequent RPL measurement. Relying on a perfect evaluation index system (EIS), QRL evaluation reveals the actual influence of rural revitalization over the affluence level and RPL of residents in the pastoral areas. Following the complete, scientific, reasonable, and operable principles, this paper refers to the relative poverty indices of Gustafsson and Sai (Ravallion and Chen 2019), Ma et al. (2019) (Ren et al. 2017), and Guanghua Wan et al. (Reynolds 1988) and constructs a QRL EIS suitable for our research samples, in the light of the actual situation of the study area. The EIS involves 12 indices of 4 categories: income and expenditure, living infrastructure, public service and social security, and eco-environment (Table 1). Among them, income, expenditure, living infrastructure, and public services have a certain impact on the quality of living standard for the residents in pastoral areas (Ravallion and Chen 2019; Ren et al. 2017; Reynolds 1988). Particularly, the e-commerce demonstration counties, Internet broadband access, distance to highway, and number of mobile phones have an explicit influence on the economic and social status of the residents. In addition, the ecological environment has a strong connection with residents’ personal life quality (Sen 1985; Huang and Cox 2016; Tafran et al. 2020).

**Table 1:** EIS, COVs, and weights of QRLs of pastoral areas

Category	Code	Index	Unit	Property	COV	Weight (%)
Income and expenditure	X1	Per capita disposable income	yuan	Positive	0.67	5.43
	X2	Education expenditure	10,000 yuan	Positive	1.24	10.09
Living infrastructure	X3	E-commerce demonstration county/banner	0–1 distribution	Positive	1.66	13.46
	X4	Number of broadband users	household	Positive	1.58	12.84
	X5	Highway mileage	km	Positive	0.72	5.83
	X6	Number of mobile phone users	10,000 households	Positive	1.38	11.22
Public service and social security	X7	Number of basic old-age insurance subscribers	10,000 people	Positive	0.88	7.15
	X8	Number of beds in medical and health organizations	each	Positive	1.35	10.94
	X9	Number of basic health insurance subscribers	10,000 people	Positive	0.75	6.11
Eco-environment	X10	Temperature	°C	Negative	0.59	4.81
	X11	Rainfall	mm	Positive	0.62	5.05
	X12	Grassland area	10,000 mu	Positive	0.87	7.07

Weights were calculated after normalization by formulas (1)-(4). COV is the coefficient of variation

Based on the QRL EIS (Table 1), a QRL evaluation model can be established as:

5 $$QRL=\sum_{\mathrm{i}=1}^{\mathrm{n}}{W}_{\mathrm{i}}\times {Y}_{\mathrm{i}}$$

#### RPL calculation

Drawing on Gustafsson and Sai (2020) (Ravallion and Chen 2019) and Ma et al. (2019) (Ravallion and Chen 2011), this paper evaluates the RPL of the pastoral areas by:

6 $$RPI=\frac{{I\mathrm{c}}_{\mathrm{i}}+{G\mathrm{c}}_{\mathrm{i}}+{F\mathrm{c}}_{\mathrm{i}}}{3}$$

7 $$PI\mathrm{c}=\left\{\begin{array}{c}\frac{{I\mathrm{c}}_{\mathrm{i}}-I\mathrm{d}}{{I\mathrm{c}}_{\mathrm{i}}},{I\mathrm{c}}_{\mathrm{i}}<I\mathrm{d}\\ 0, {I\mathrm{c}}_{\mathrm{i}}>I\mathrm{d}\end{array}\right\}$$

8 $$PG\mathrm{c}=\left\{\begin{array}{c}\frac{{P\mathrm{c}}_{\mathrm{i}}-P\mathrm{d}}{P\mathrm{ci}}, {P\mathrm{c}}_{\mathrm{i}}<P\mathrm{d}\\ 0, {P\mathrm{c}}_{\mathrm{i}}>IP\mathrm{d}\end{array}\right\}$$

9 $$PF\mathrm{c}=\left\{\begin{array}{c}\frac{{F\mathrm{c}}_{\mathrm{i}}-F\mathrm{d}}{F\mathrm{ci}},{F\mathrm{c}}_{\mathrm{i}}<F\mathrm{d}\\ 0, {F\mathrm{c}}_{\mathrm{i}}>F\mathrm{d}\end{array}\right\}$$

where $RPL$, $PI\mathrm{c}$, $PG\mathrm{c}$, and $PF\mathrm{c}$ are relative poverty level, the gap of rural per capita income, the gap of rural per capita GDP, and gap of rural per capita poverty alleviation fund, respectively; ${I\mathrm{c}}_{\mathrm{i}}$, ${G\mathrm{c}}_{\mathrm{i}}$, and ${F\mathrm{c}}_{\mathrm{i}}$ are per-capita income, GDP, and poverty alleviation fund of county/banner *i*, respectively; $I\mathrm{d}$, $P\mathrm{d}$, and *F*d are per capita income, GDP, and poverty alleviation fund of Inner Mongolia, respectively. The greater the $RPI$, the higher the RPL of an area.

#### Composite RPL calculation

Based on the formulas of QRL and RPL, this paper uses the poverty object identification model of Gustafsson and Sai (Ravallion and Chen 2019) to judge whether the 18 pastoral areas in Inner Mongolia are relatively poor areas:

10 $$CPL=\sqrt{{QRL}^{2}+{RPI}^{2}-\frac{{\left({QRL}^{2}-QRL\times RPI\right)}^{2}}{{2QRL}^{2}}}$$

## Results Analysis

### Spatial layout of QRLs

#### QRL calculation

##### (1) COVs and weights

The data were normalized by formulas (1)–(2). For the lack of space, the normalized data are not presented here. The COVs and weights of the QRLs of the pastoral areas were computed by formulas (3) and (4), respectively. The calculation results are recorded in Table 1. The COV reflects the dispersion of data. The greater the COV, the more imbalanced the distribution of QRLs among the pastoral areas. The COVs of five indices, namely, education expenditure (1.24), e-commerce demonstration county/banner (1.66), number of broadband users (1.58), number of mobile phone users (1.38), and number of beds in medical and health organizations (1.35), were relatively large (> 1), indicating that the original data of the five indices are rather dispersed. The weights of the five indices, namely, e-commerce demonstration county/banner (13.46%), number of broadband users (12.84%), number of mobile phone users (11.22%), number of beds in medical and health organizations (10.94%), and education expenditure (10.09%), were relatively great, revealing the major impacts of the five indices on QRL; i.e., the five indices play a critical role in improving QRL. Since the printing and release of the Outline of Digital Countryside Development Strategy in May 2019, Inner Mongolia has seriously implemented the strategy. The construction of digital countryside is the strategic direction of rural revitalization and is closely related to the living quality of farmers and herdsmen. Therefore, the number of broadband users, number of mobile phone users, and e-commerce demonstration county/banner are the gist of the construction of digital countryside and important drivers of QRL.

##### (2) Composite scores of QRLs in pastoral areas

**Fig. 1:**
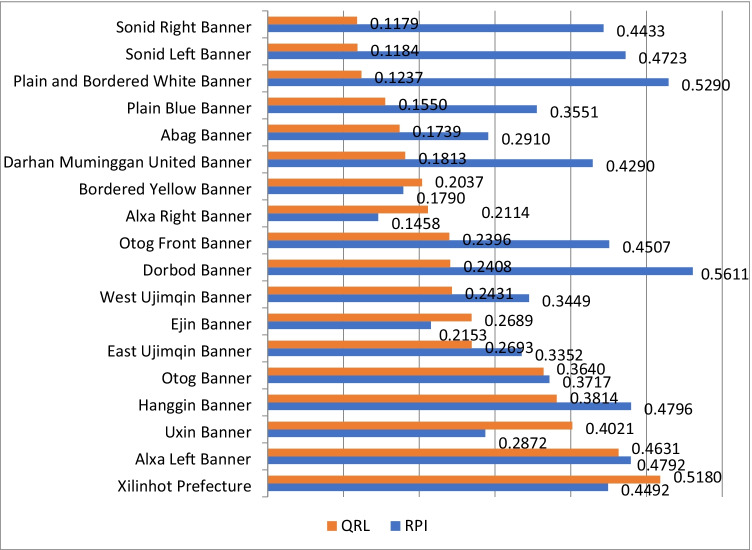
QRLs and RPLs of pastoral areas in Inner Mongolia

The QRLs of the 18 pastoral counties/banners were measured by formula (5). The higher the score, the greater the QRL. The results show that the QRLs of pastoral areas in Inner Mongolia were unbalanced. The mean score of QRLs was 0.2598, far smaller than 1. Eleven (61.11%) of the counties/banners had a QRL smaller than the mean score. Xilinhot Prefecture achieved the highest QRL (0.5180). Alxa Left Banner (0.4631), Uxin Banner (0.4021), Hanggin Banner (0.3814), and Otog Banner (0.3640) realized relatively high QRLs. Sonid Right Banner ended up with the lowest QRL (0.1179), trailing the top-ranking county/banner by 0.4001 (Fig. 1). The above analysis shows that the pastoral areas in Inner Mongolia have relatively low QRLs, and the counties/banners differed greatly in QRL. The reason lies in the wide gaps between the counties/banners in income and expenditure, public service and social security, living infrastructure, and eco-environment, which reflect their disparity in geographical location, special industries, and natural resources. These four factors are important indicators of the living quality in a region.

#### Spatial features of QRLs of pastoral areas

With the aid of ArcGIS 10.8, the QRLs of the pastoral areas were identified by the natural breakpoint method, and the pastoral areas were divided into the following levels: strongly low (< 0.123701), slightly low (0.123702–0.181337), medium (0.181338–0.243127), slightly high (0.243128–0.269267), and strongly high (> 0.269268). On this basis, the spatial layout of QRLs of pastoral areas in Inner Mongolia was visualized, revealing the spatial landscape and role of the QRL of each county/banner.

**Fig. 2:**
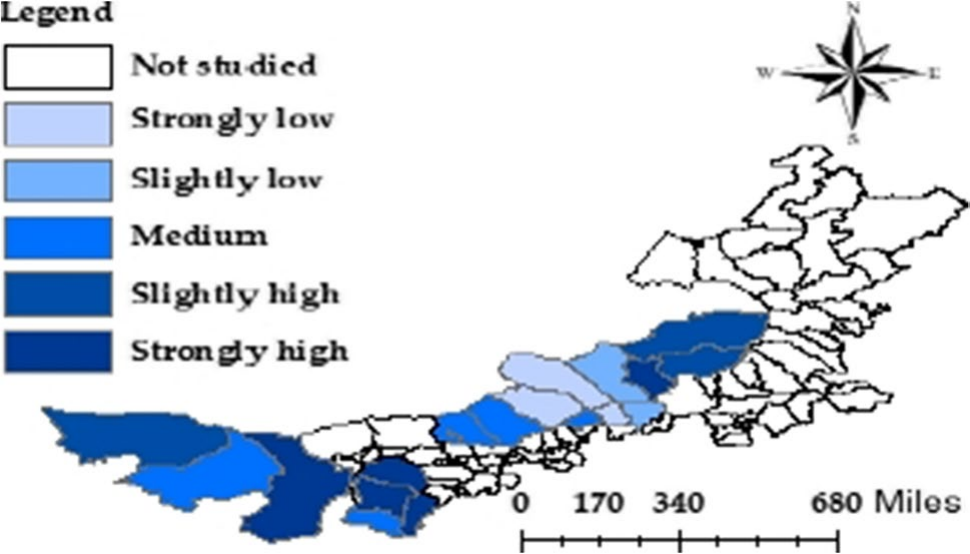
Layout of QRLs of pastoral areas in Inner Mongolia

As shown in Fig. 2, there were significant spatial differences between the pastoral areas in Inner Mongolia in terms of QRL: the western areas were stronger than the central areas. High QRL counties/banners are mainly concentrated in the western region. The QRLs in the western region where at least the medium level. In the central region, the QRLs were very fragmented, falling onto all five levels. Therefore, western pastoral areas generally had a higher QRL than their central counterparts. In terms of QRL, Xilinhot Prefecture led the central region, and Alxa Left Banner led the western region.

It can also be seen from Fig. 2 that six pastoral areas belonged to relatively and strongly low levels of QRL (33.33%), five belonged to the medium level (27.78%), and seven belonged to relatively and strongly high levels (38.89%). The mean QRL (0.2598) of the pastoral areas in Inner Mongolia suggests that the pastoral areas in central and western Inner Mongolia have a poor quality of life, although getting out of absolute poverty. The natural breakpoint results show that nearly 62% of pastoral areas in Inner Mongolia face a low to medium QRL. Therefore, the living quality of the farmers and herdsmen should be improved substantially to avoid large-scale returning to poverty and to facilitate the realization of common prosperity.

### Spatial layout of RPLs of pastoral areas

#### RPLs of pastoral areas

In 2020, China announced that it had built a moderately prosperous society in all respects and eliminated absolute poverty. However, relative poverty was expected to linger for quite a long time. The solution to relative poverty is the key to dock poverty eradication with rural revitalization. Thus, it is of great practical significance to measure and analyze RPL. This paper computes the RPL scores of the pastoral areas in central and western Inner Mongolia by formulas (6)–(9). The higher the score, the greater the RPL.

As shown in Fig. 1, the pastoral areas in central and western Inner Mongolia differed significantly in RPL. The mean score of RPL stood at 0.3788. Nine counties/banners (50%) had an RPL greater than the mean. Dorbod Banner (0.5611) achieved the highest RPL. Relatively high scores were realized by Plain and Bordered White Banner (0.5290), Hanggin Banner (0.4796), Alxa Left Banner (0.4792), and Sonid Left Banner (0.4723). Relatively low RPLs were witnessed by Bordered Yellow Banner (0.1790), Ejin Banner (0.2153), and Uxin Banner (0.2872). The lowest RPL belonged to Alxa Right Banner (0.1458), 0.4153 smaller than the RPL of the top-ranking Dorbod Banner. The above analysis shows that the pastoral areas in central and western Inner Mongolia have a huge difference in the RPL. The result agrees well with what is reflected by QRLs.

#### Spatial features

With the aid of ArcGIS 10.8, the RPLs of the pastoral areas were identified by the natural breakpoint method, and the pastoral areas were divided into the following levels: strongly low (< 0.215323), slightly low (0.215324–0.291022), medium (0.291023–0.371750), slightly high (0.371751–0.479568), and strongly high (> 0.567052). On this basis, the spatial layout of RPLs of pastoral areas in Inner Mongolia was visualized, revealing the spatial landscape of the RPL of each county/banner.

**Fig. 3:**
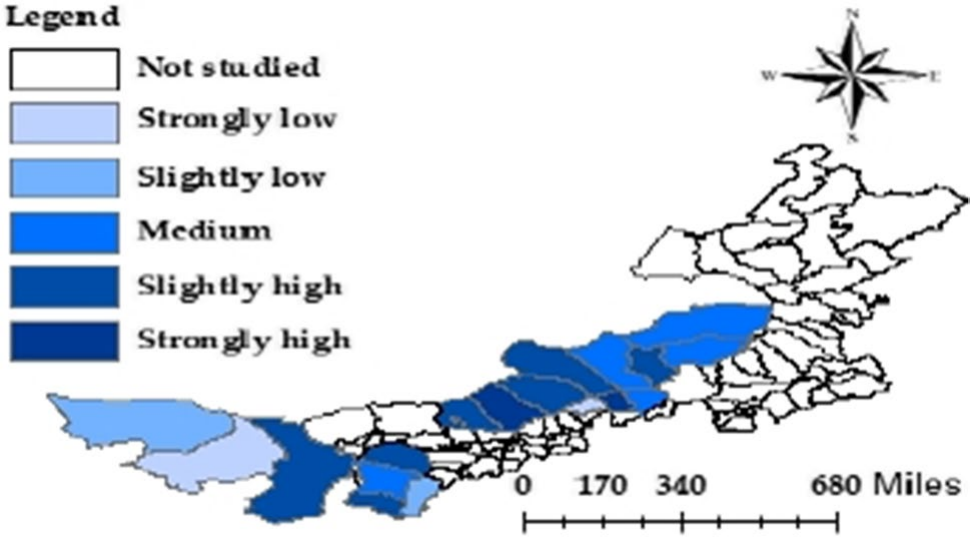
Layout of RPLs of pastoral areas in Inner Mongolia

As shown in Fig. 3, contrary to the spatial layout features of QRLs, the central pastoral areas in Inner Mongolia had stronger RPLs than the eastern ones. High RPL counties/banners are mostly clustered in the central region. Alxa Left Banner and Dorbod Banner were the leaders of RPL in the western and central regions, respectively. The spatial layout of RPLs is relatively reasonable in the central region: the RPLs decreased gradually from Dorbod Banner.

It can also be seen from Fig. 3 that 50% of the pastoral areas belonged to strongly and slightly high levels of RPL. Although China has eradicated absolute poverty, the RPLs of pastoral areas in Inner Mongolia remains high. To dock poverty eradication with rural revitalization, the key is to measure the RPL and identify relatively poor areas.

### Identification of relatively poor areas

The composite RPLs of the 18 pastoral areas were computed by formula (10). The pastoral areas with relatively high composite RPLs face a high risk of returning to poverty. The mean composite RPL was calculated to preliminarily identify the pastoral areas with a significant RPL. In addition, the index systems of QRL and RPL were combined to mine the deep-seated reasons for the high RPL. On this basis, proper suggestions were given for suppressing the risk of poverty-returning in these counties/banners.

The results show that eight counties/banners (44.44%) had a high composite RPL (greater than the mean of 0.4515), including Xilinhot Prefecture, Alxa Left Banner, Hanggin Banner, Dorbod Banner, Otog Banner, Otog Front Banner, Uxin Banner, and Plain and Bordered White Banner. These counties/banners have a low income, backward infrastructure, poor public service and social security, and harsh eco-environment. Xilinhot Prefecture (0.6839) achieved the highest composite RPL (0.6839), leading the worst performer Alxa Right Banner (0.2526) by 0.4313. The above analysis shows that nearly 45% of the pastoral areas in central and western Inner Mongolia face serious relative poverty and a high risk of returning to poverty. These counties/banners need to improve the governance of relative poverty in rural pastoral areas and treat the reduction of RPL as the top priority.

With the aid of ArcGIS 10.8, the composite relatively poor areas were identified by the natural breakpoint method, and the pastoral areas were divided into the following levels: strongly low (< 0.270575), slightly low (0.270576–0.360725), medium (0.360726–0.431565), slightly high (0.431566–0.567051), and strongly high (> 0.567052). On this basis, the areas with strong and slightly high RPLs were defined as composite relatively poor areas.

**Fig. 4:**
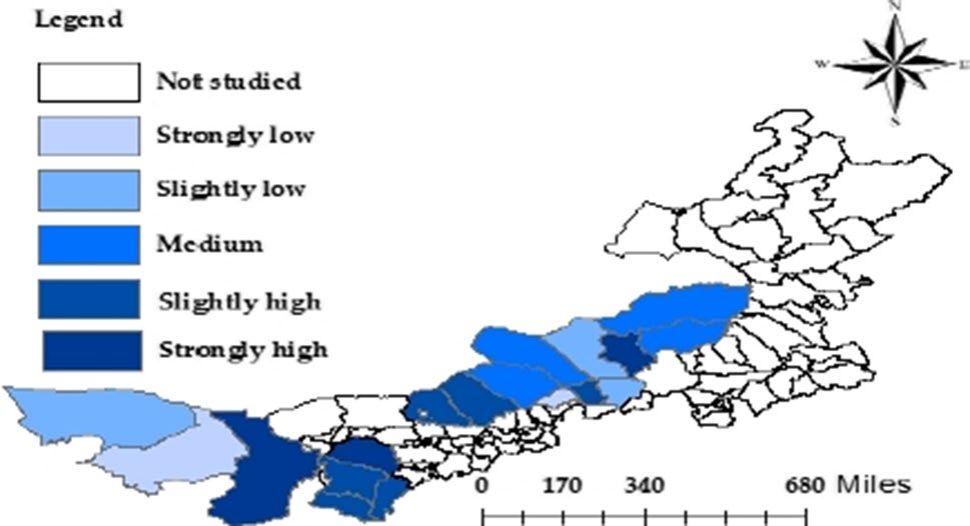
Layout of composite RPLs of pastoral areas in Inner Mongolia

As shown in Fig. 4, eight areas (44.44%), namely, Xilinhot Prefecture, Alxa Left Banner, Hanggin Banner, Dorbod Banner, Otog Banner, Otog Front Banner, Plain and Bordered White Banner, and Uxin Banner, are relatively poor areas. This further confirms that these eight areas are composite relatively poor areas. It can also be seen from Fig. 4 that the composite relatively poor counties/banners clustered clearly in space, mainly in the western region. Thus, the pastoral areas in the western region generally have a high composite RPL. In addition, the counties/banners with a high composite RPL were close to each other, in both western and central regions. In the western region, the composite relatively poor counties/banners spanned continuously from Alxa Left Banner to Otog Front Banner. In the central region, the composite RPL decreased from Dorbod Banner and Plain and Bordered White Banner to the outside.

The high composite RPL means a high risk of returning to poverty. An important reason for the high composite RPL lies in the frequent occurrence of weather disasters. For example, most residents in pastoral areas, especially those being lifted out of poverty, in Alxa Left Banner of western Inner Mongolia and Otog Banner, Hanggin Banner and Uxin Banner of Ordos Prefecture, lack diverse income sources. They mainly live on animal husbandry and crop farming. According to the data on QRL indices, the annual mean temperatures of Alxa Left Banner, Otog Banner, Hanggin Banner, and Uxin Banner are 4.23 °C above the annual mean temperature of the study area. As a result, these pastoral areas suffer frequently from draughts and wind damages, which seriously constrain farming and animal husbandry and amplify the risk of returning to poverty. In addition, Fig. 3 indicates that Otog Banner, Hanggin Banner, and Uxin Banner of Ordos Prefecture had a composite RPL slightly lower than that of Alxa Left Banner. This is because the counties/banners of Ordos Prefecture boast a stronger composite economic strength than Alxa Left Banner, and thus a greater ability to resist risks. This is testified by the high-income levels of all counties/banners of Ordos Prefecture and the fact that all these counties/banners are recognized as e-commerce demonstration counties/banners. The residents in the pastoral areas of central Inner Mongolia are also troubled by weather disasters. The annual mean temperatures of Dorbod Banner and Plain and Bordered White Banner were 4.23 °C below the annual mean temperature of the study area. Hence, the central region is not only hit by droughts and wind damage but also the snowstorms brought by the low temperature.

Apart from the poverty-returning risk induced by weather disasters, low education levels and poor medical conditions are important drivers of composite RPLs in these areas. For example, the medical and health organizations in Plain and Bordered White Banner and Dorbod Banner of central Inner Mongolia have 636 fewer beds than the mean value. In 2020, the education expenditure of Plain and Bordered White Banner was only 97.21 million yuan, which fell short of the mean value in the study area.

## Discussion

Solving the relative poverty problem is an important task that consolidates the results of poverty eradication and facilitates rural revitalization. The RPL has a high diversity and stems from different causes in different regions and groups (UNDP 2010; Plakhin 2014; Li et al. 2016). It can be affected by factors like income and expenditure, living infrastructure, public service and social security, and eco-environment. These factors change with time and space, making relative poverty more diverse. For many reasons, some relatively poor areas and groups are very likely to return to poverty (Guanghua et al. 2018), especially the special pastoral areas (Aaberge and Brandolini 2015). Therefore, the relatively poor areas and objects that may return to poverty must be identified timely and accurately. Otherwise, it would be impossible to dock poverty eradication with rural revitalization or overcome the thorny problem of relative poverty.

From the angle of spatial layout, this paper computes QRL and then measures RPL. With the aid of ArcGIS 10.8, composite relatively poor areas were identified by the natural breakpoint method. Compared with the previous research, this paper measures the RPLs of special pastoral areas and identifies composite relatively poor areas, from the perspective of spatial layout. The methodology and perspective are innovative, and the results are relatively reliable. The research data were county-level data from statistical yearbooks. The variable selection and data processing may be limited to a certain degree. However, the selected indices are very representative (Ren et al. 2017) and work effectively in measuring RPL and identifying composite relatively poor areas. Because the data are complex and difficult to obtain, this paper considers the dynamic changes in relative poverty and decides to compute QRL before measuring RPL. In the previous studies, QRL and subjective well-being were purely dependent on subjective feelings (Wang et al. 2021; Wu et al. 2019; Yang et al. 2019). QRL also depends on objective conditions like income and expenditure, living conditions and cultural life, rural infrastructure, public service and social security, and eco-environment (Zeng et al. 2021). It is a comprehensive metric of the living standard of a region. Therefore, this paper objectively measures RPLs according to the QRLs of the pastoral areas and identifies composite relatively poor areas, providing a reference for decision-makers to govern relative poverty in pastoral areas. Finally, several practical suggestions were proposed to prevent a large-scale return to poverty, from the angle of the poverty-returning warning mechanism.

The composite identification of relative poverty is systematic and dynamic. Although our research data, indices, and methods were selected after thorough consideration, only 18 pastoral areas were studied in central and western Inner Mongolia, China, due to the limitations of data statistics and availability, most of the index data in this paper are positive indicators, while there is only one negative indicator. Therefore, it is necessary to introduce more negative indicators in future research to establish a more scientific and efficient quality of life index system and then measure the living level comprehensively and objectively. Of all pastoral areas in Inner Mongolia, 45.4% belong to the eastern region, which differs from the central and western regions in terms of QRL, PRL, and composite relatively poor areas. In addition, future research will compare the RPLs between the three regions and measure the RPLs with the objective and subjective indices of QRL.

## Conclusions and suggestions

### Conclusions

To explore the composite relatively poor pastoral areas in Inner Mongolia, this paper draws on the spatial layout theory to evaluate QRL and measure RPL and then identify the areas with a high composite RPL. The main conclusions are as follows:

Firstly, the QRLs of pastoral areas in Inner Mongolia were unbalanced. The mean score of QRLs was 0.2598, far smaller than 1. Eleven (61.11%) of the counties/banners had a QRL smaller than the mean score. On the spatial layout of QRLs, the western areas were stronger than the central areas. High QRL counties/banners are mainly concentrated in the western region. In the central region, the QRLs were very fragmented, falling onto all five levels. Although national absolute poverty problem has been resolved, the result of the natural breakpoint indicated that the quality of life for grassland and pastoral areas is still at a medium and low level (about 62%), and there is still room to improve the quality of life for local people.

Secondly, the pastoral areas in central and western Inner Mongolia differed significantly in RPL. The mean score of RPL stood at 0.3788. Nine counties/banners (50%) had an RPL greater than the mean. Contrary to the spatial layout features of QRLs, the central pastoral areas in Inner Mongolia had stronger RPLs than the eastern ones. High RPL counties/banners are mostly clustered in the central region. The spatial layout of RPLs is relatively reasonable in the central region: the RPLs decreased gradually from Dorbod Banner. Our results pointed out that the relative poverty level of grassland pastoral areas is still high in Inner Mongolia.

Finally, nearly 45% of the pastoral areas in central and western Inner Mongolia face serious relative poverty and high risk of returning to poverty. Eight counties/banners (45%) were identified as high composite relative poverty areas, namely, Xilinhot Prefecture, Alxa Left Banner, Hanggin Banner, Dorbod Banner, Otog Banner, Otog Front Banner, Plain and Bordered White Banner, and Uxin Banner. From spatial layout, the composite relatively poor counties/banners clustered clearly, mainly in the western region. The frequent occurrence of weather disasters is the most important factor causing relative poverty. In addition, low-level education and lack of advanced medical facilities also have a significant impact.

### Suggestions

Based on the above conclusions, the following suggestions were presented:

1. Prepare precision strategies to increase the income of herdsmen, according to the local situation.
  On the one hand, put efforts into the development of the digital countryside and intelligent agriculture, carry out scientific analysis and planning of farming and animal husbandry in the pastoral areas, provide technical and market supports to herdsmen, and transform the areas with the power of science and technology. Besides, diversify the income sources of herdsmen, increase their income, and mitigate the risk induced by the limited livelihood means.
  On the other hand, construct an industrial belt of characteristic farming and animal husbandry products based on special industries, build regional public brands of green farming and animal husbandry products from pastoral areas, strengthen the connection between industrial chain and supply chain, promote the cluster development of special industries, and improve the overall economy of the pastoral areas.
2. Develop and deploy living infrastructure reasonably.
  Given the low population and sparse houses in the pastoral areas, optimize the residency policy, guide the population to advantageous or convenient areas, and accelerate the transfer of farmers and herdsmen into city dwellers. In addition, improve the interconnection level of infrastructure; build integrated transportation system; facilitate the free flow of people, materials, and information; and support the integrated development.
  Step up the construction of the agricultural information network to provide herdsmen with accurate and timely agricultural information and enhance the interconnection between pastoral areas. Rely on the construction of e-commerce demonstration counties/banners and digital countryside, integrate e-commerce into comprehensive rural demonstration and digital countryside, speed up the establishment of a standard system that adapts to the development of agricultural products, and promote the fast development of digital countryside and the interconnection between agriculture and commerce.
3. Divert more financial resources to public service and social security and provide herdsmen in the pastoral areas with more comprehensive and convenient services.
  Combined with the features of pastoral areas, establish an equalization system for basic public service, expand high-quality medical resources, balance the resource distribution in the region, and realize the comprehensive coverage of basic old-age and medical insurances, thereby standardizing and facilitating public services, promoting urban and rural integration, and building a new pattern of rural revitalization. This is an effective way to improve the living standards and quality of the herdsmen.
4. Based on the Two Mountains Theory (i.e., lucid waters and lush mountains are invaluable assets), enhance the eco-environmental protection awareness of herdsmen.
  In the pastoral areas, herdsmen need to transform their livelihood means, owing to frequent droughts, severe grassland damages, and the green for green project. The pastoral areas should be planned according to the development goals of rural revitalization, while maintaining the rural spatial layout, where human and nature are organically integrated.
  Firstly, strengthen the environmental awareness of herdsmen to prevent excessive grazing and ensure forage-animal balance. Protect the key ecological zones and improve the life of the herdsmen switching to another livelihood means by guiding the shift of herdsmen in key ecological zones and poor production zones of the grassland to other sectors and perfecting the ecological compensation mechanism.
  Secondly, improve the policies on domestic garbage treatment of agriculture and pastoral areas, implement garbage sorting, manage straw combustion and excessive use of pesticides and plastics, and reduce environmental pollution.
  Finally, the government should increase green procurement, promote green products, strengthen the willingness of herdsmen to produce green products, and accelerate the development of the green economy.
  In addition to economic development, introduce clean energy, rationalize the layout of advantageous special industries, promote the joint construction and protection of ecology, and work together to pursue low-carbon, green, cyclical development, such as to achieve indusial prosperity, ecological livability, rural civilization, and effective governance.
5. Establish and improve the warning mechanism for poverty-returning.

In terms of recognizing potential poverty-returning objects, combine data identification with intra-village mutual evaluation, clarify the screening standards for these objects, and improve the mechanism of data collection and sharing. Preliminarily recognize the potential objects based on big data, and integrate the results of data identification with those of mutual evaluation. On this basis, select the potential poverty-returning objects, and analyze the main causes of poverty-returning.

In terms of handling potential poverty-returning, develop the countermeasures according to the level and region. Use a regional grading standard to divide the levels, and adopt different measures for different levels. For example, perform ideological education for the potential poverty-returning households, who are not active in poverty alleviation; provide those active in poverty alleviation with policy support, technical guidance, and market guidance.

In addition, pay attention to risky events of poverty-returning with the aid of big data, including the frequency of risky events in the insurance coverage, carry out the early intervention, and reduce the potential risk of poverty-returning.

## Data Availability

Data will be provided on request.
